# Analysis of Flavonoid Metabolites in Buckwheat Leaves Using UPLC-ESI-MS/MS

**DOI:** 10.3390/molecules24071310

**Published:** 2019-04-03

**Authors:** Jing Li, Pu Yang, Qinghua Yang, Xiangwei Gong, Hongchi Ma, Ke Dang, Guanghua Chen, Xiaoli Gao, Baili Feng

**Affiliations:** College of Agronomy, Northwest A & F University, State Key Laboratory of Crop Stress Biology for Arid Areas, Yangling, Xianyang 712000, China; lijing1993@nwafu.edu.cn (J.L.); yangpu@nwsuaf.edu.cn (P.Y.); 2016060037@nwsuaf.edu.cn (Q.Y.); gxw199308@163.com (X.G.); mhcnzb@163.com (H.M.); dangke4718@163.com (K.D.); ghchen2017@163.com (G.C.); gao2123@nwsuaf.edu.cn (X.G.)

**Keywords:** tartary buckwheat, common buckwheat, flavonoid metabolites, PCA, OPLS-DA, UPLC-ESI-MS/MS

## Abstract

Flavonoids from plants are particularly important in our diet. Buckwheat is a special crop that is rich in flavonoids. In this study, four important buckwheat varieties, including one tartary buckwheat and three common buckwheat varieties, were selected as experimental materials. The total flavonoid content of leaves from red-flowered common buckwheat was the highest, followed by tartary buckwheat leaves. A total of 182 flavonoid metabolites (including 53 flavone, 37 flavonol, 32 flavone C-glycosides, 24 flavanone, 18 anthocyanins, 7 isoflavone, 6 flavonolignan, and 5 proanthocyanidins) were identified based on Ultra Performance Liquid Chromatography–Electrospray Ionization–Tandem Mass Spectrometry (UPLC-ESI-MS/MS) system. Through clustering analysis, principal component analysis (PCA), and orthogonal signal correction and partial least squares-discriminant analysis (OPLS-DA), different samples were clearly separated. Considerable differences were observed in the flavonoid metabolites between tartary buckwheat leaves and common buckwheat leaves, and both displayed unique metabolites with important biological functions. This study provides new insights into the differences of flavonoid metabolites between tartary buckwheat and common buckwheat leaves and provides theoretical basis for the sufficient utilization of buckwheat.

## 1. Introduction

Buckwheat is an annual eudicot plant belonging to the family Polygonaceae, genus *Fagopyrum* [1]. Tartary buckwheat (*Fagopyrum tataricum* (L.) Gaertn) and common buckwheat (*Fagopyrum esculentum* Moench) are the two main species of buckwheat [2], which are considered as alternative crops or minor cereals and are popular food in Asia and Europe [3,4]. Tartary buckwheat and common buckwheat are traditionally regarded as medicinal and food homologous crops, because their grains are characterized by high contents of starch and dietary fibre, and protein with an advantageous amino acid composition. Furthermore, they are the only pseudocereals that contain rutin, an important flavonoid [5,6,7]. Flavonoids is an important group of plant secondary metabolites and include anthocyanins, flavanes, flavones, flavanones, flavonols, and chalcones [8]. Previous studies have shown that flavonoids are involved in many biological functions and have important health-related roles, such as anti-oxidative, anti-diabetic, anti-inflammatory, and anti-hypertensive activities [9,10,11,12,13,14]. Flavonoids from plants are particularly important in our diet because flavonoids cannot be synthesized by humans and animals [15,16].

Currently, tartary buckwheat and common buckwheat have been made into a series of consumer foods, such as pancakes, bread, noodles, and tea [17,18]. However, only their grains are used in food production. The accumulation of flavonoids is tissue-specific [19]. The flavonoid content in leaves is higher than that in grains of tartary buckwheat and common buckwheat, and the rutin content of common buckwheat leaves during flowering and seed formation period are even higher than that of flowers [20]. Moreover, buckwheat is a eudicot and has considerable leaf biomass, thus, the buckwheat leaf has great potential for utilization. The rutin content in tartary buckwheat grains and sprouts are higher than that in common buckwheat [16,21]; however, compared with tartary buckwheat, common buckwheat is rich in luteolin and apigenin, which are two types of flavonoids with great antioxidant activity [2,22]. Therefore, a comprehensive investigation of flavonoid metabolites in tartary buckwheat and common buckwheat leaves is highly important. Stojilkovski et al. [23] compared the contents of fagopyrin, rutin, and quercetin in common tartary and cymosum buckwheat, and the common buckwheat leaves displayed high levels of fagopyrin. Investigations on the various flavonoid metabolites in tartary buckwheat and common buckwheat leaves have not been done. In this study, an integrated detection system Ultra Performance Liquid Chromatography–Electrospray Ionization–Tandem Mass Spectrometry (UPLC-ESI-MS/MS) coupled with clustering analysis, principal component analysis (PCA), and orthogonal signal correction and partial least squares-discriminant analysis (OPLS-DA) were used to investigate the differences in flavonoid metabolites of tartary buckwheat and common buckwheat leaves, to provide theoretical basis for utilizing buckwheat leaves.

## 2. Results

### 2.1. Determination of Total Flavonoid Content

**Figure 1:**
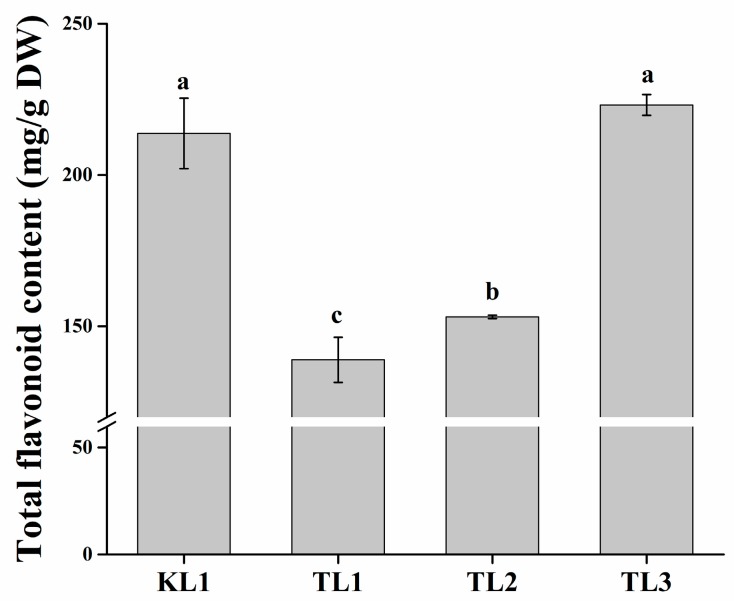
Total flavonoid content of tartary buckwheat and common buckwheat leaves. The lower-case letters above the histogram indicated the statistical significance at the level of 0.05 (*p* < 0.05).

In this study, one tartary buckwheat variety “Xi-nong 9940” (KL1) and three common buckwheat varieties “Bei-zao-sheng” (TL1), “Xi-nong 9978” (TL2), and “Xi-nong 1351” (TL3) were selected as experimental varieties. The total flavonoid contents in the leaves of the four buckwheat varieties were investigated (Figure 1). The flavonoid content of TL3 was slightly higher than that of KL1 but the difference was not significant (*p* < 0.05), whereas it was significantly higher (*p* < 0.05) than that of TL1 and TL2, reaching 223.04 mg/g DW. The flavonoid content of the leaves from tartary buckwheat was the second highest at 213.66 mg/g DW. Moreover, the flavonoid content of TL2 was significantly higher (*p* < 0.05) than that of TL1. TL1 had the lowest flavonoid content of 138.95 mg/g DW.

### 2.2. Metabolic Profiling

**Figure 2:**
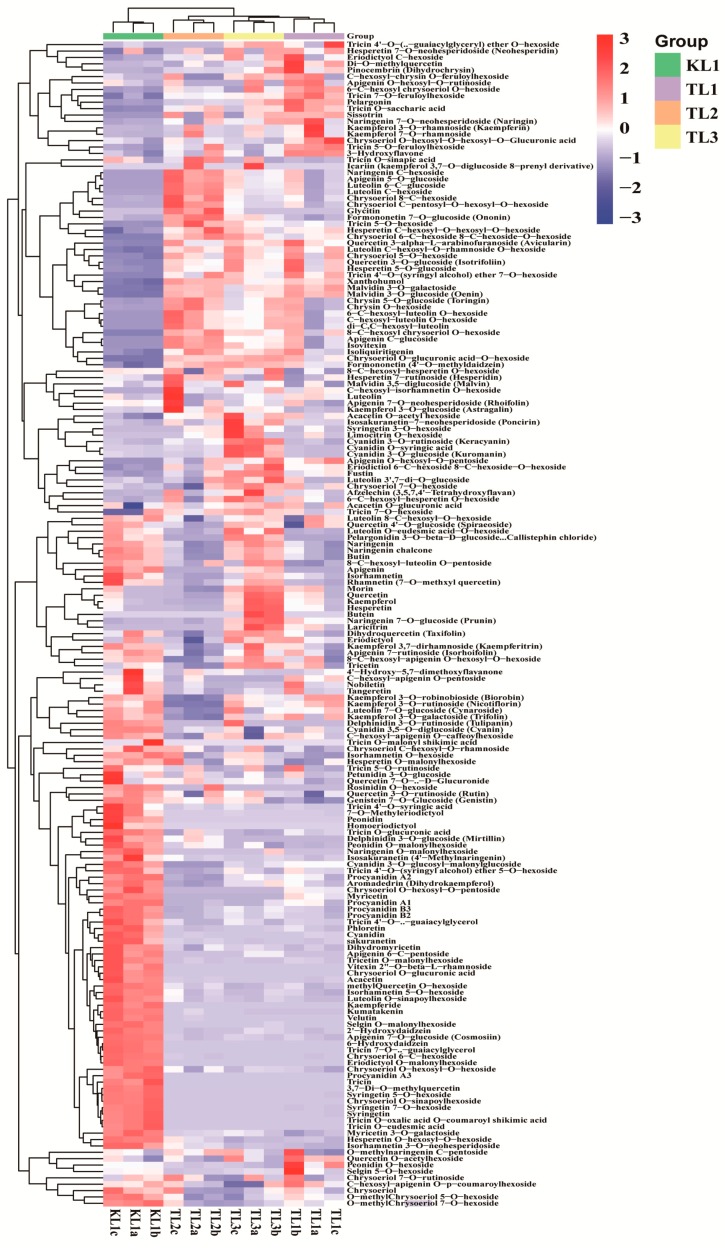
Clustering heat map of all flavonoid metabolites. The metabolite content data was normalized by maximum difference normalization method. Each sample was represented by a column, and each metabolite was represented by a row. The abundance of each metabolite was represented by a bar with specific color. The up-regulated and down-regulated metabolites were expressed with different shade colors of red and blue, respectively. With the increase in the abundance value, the color of the bar presented from blue to red. When the abundance value was 0, the color of bar was white, as shown in the bar at the upper right.

The flavonoid metabolites of the leaves from four buckwheat varieties were investigated based on UPLC-ESI-MS/MS and databases. In this study, 182 flavonoid metabolites were identified, including 53 flavone, 37 flavonol, 32 flavone C-glycosides, 24 flavanone, 18 anthocyanins, 7 isoflavone, 6 flavonolignan, and 5 proanthocyanidins (Supplementary Table S1). All flavonoid metabolites of the samples are shown in Figure 2 as a heat map after homogenization. In the heat map, the content of flavonoid metabolites in the KL1 contrasted with TL1, TL2, and TL3 varied greatly, whereas the contents of flavonoid metabolites among TL1, TL2, and TL3 were basically consistent. This finding was demonstrated by clustering analysis of the samples and showed that the tartary buckwheat was clearly distinguished from three common buckwheat varieties. Meanwhile, by clustering all flavonoid metabolites, more than half of the flavonoid metabolites content in KL1 were higher than those in TL1, TL2, and TL3.

### 2.3. Differential Flavonoid Metabolite Analysis Based on PCA

**Figure 3:**
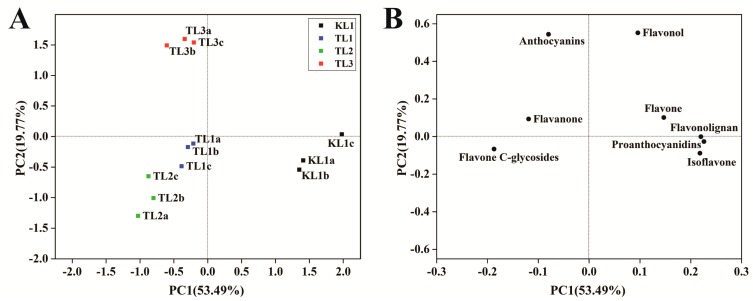
Differential flavonoid metabolite analysis on the basis of principal component analysis (PCA). (**A**) PCA score plot (**B**) PCA loading plot.

PCA uses several principal components to reveal the internal structure between multiple variables. In this study, two principal components, PC1 and PC2, were extracted and were 53.49% and 19.77%, respectively; moreover, the cumulative contribution rate reached 73.26%. In the PCA score plot (Figure 3A), KL1, TL1, TL2, and TL3 were clearly separated, and the repeated samples were compactly gathered together, thus indicating that the experiment was reproducible and reliable. The distinct separation of KL1 from TL1, TL2, and TL3 indicated a large difference between them. In the PCA loading plot (Figure 3B), different classes of flavonoid metabolites were distributed in different quadrants. By comparing the score plot, it was found that anthocyanins, flavanones and flavone C-glycosides contributed greatly to TL1, TL2, and TL3, and other flavonoids contributed to KL1. The difference in the contents of these flavonoids might be responsible for the differences between tartary buckwheat and common buckwheat.

### 2.4. Differential Flavonoid Metabolite Analysis Based on OPLS-DA

**Figure 4:**
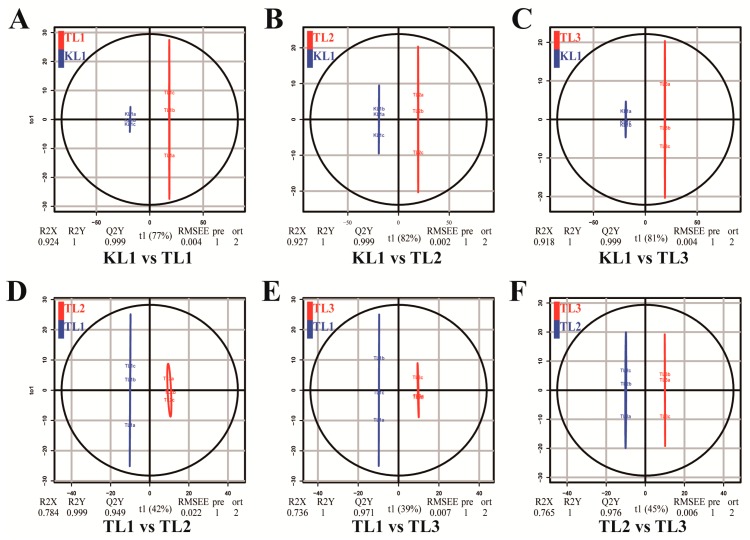
Differential flavonoid metabolite analysis on the basis of orthogonal signal correction and partial least squares-discriminant analysis (OPLS-DA). (**A**–**F**) OPLS-DA model plots for the comparison group KL1 vs TL1, KL1 vs TL2, KL1 vs TL3, TL1 vs TL2, TL1 vs TL3, and TL2 vs TL3, respectively.

OPLS-DA is an efficient method for finding differential metabolites because it can maximize the distinction between groups. Q^2^ is an important parameter for evaluating the model in OPLS-DA. The Q^2^ value greater than 0.9 indicated an excellent model. In this study, the OPLS-DA model compared the flavonoid metabolite content of the samples in pairs to evaluate the difference between KL1 and TL1 (R^2^X = 0.924, R^2^Y = 1, Q^2^ = 0.999; Figure 4A), between KL1 and TL2 (R^2^X = 0.927, R^2^Y = 1, Q^2^ = 0.999; Figure 4B), between KL1 and TL3 (R^2^X = 0.918, R^2^Y = 1, Q^2^ = 0.999; Figure 4C), between TL1 and TL2 (R^2^X = 0.784, R^2^Y = 0.999, Q^2^ = 0.949; Figure 4D), between TL1 and TL3 (R^2^X = 0.736, R^2^Y = 1, Q^2^ = 0.971; Figure 4E), and between TL2 and TL3 (R^2^X = 0.765, R^2^Y = 1, Q^2^ = 0.976; Figure 4F). The Q^2^ values of all comparison groups exceeded 0.9, thus demonstrating that these models were stable and reliable and could be used to further screen for differential flavonoid metabolites.

### 2.5. Differential Flavonoid Metabolite Screening, Functional Annotation, and Enrichment Analysis

**Figure 5:**
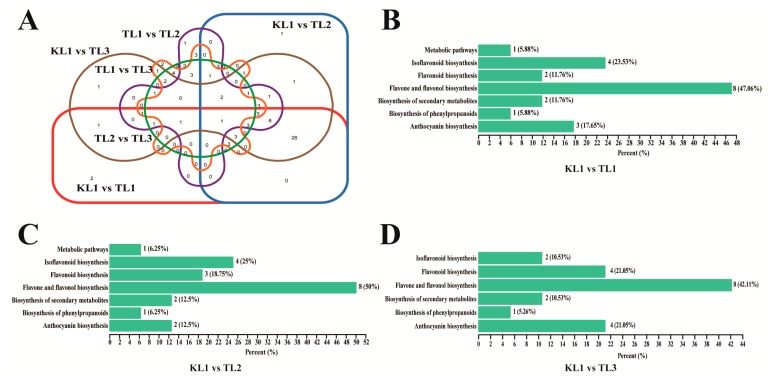
Venn diagram of differential flavonoid metabolites for each comparison group and KEGG classification results. (**A**) Venn diagram showed the overlapping and unique differential metabolites amongst the comparison groups. Circles with different shapes and different colors of red, blue, brown, purple, orange, and green represented different comparison groups of KL1 vs TL1, KL1 vs. TL2, KL1 vs. TL3, TL1 vs. TL2, TL1 vs. TL3, and TL2 vs. TL3, respectively. (**B**–**D**) The differential metabolites KEGG classification of the comparison group KL1 vs. TL1, KL1 vs. TL2, and KL1 vs. TL3, respectively.

Differential flavonoid metabolites were screened for each comparison groups by combining the fold change and variable importance in project (VIP) values of the OPLS-DA model. The criteria for screening included the fold change value of ≥ 2 or ≤ 0.5 and the VIP value of ≥ 1. The screening results are shown in Supplementary Table S2. Concisely, there were 53 significantly different flavonoid metabolites between KL1 and TL1 (35 down-regulated, 18 up-regulated), 61 between KL1 and TL2 (39 down-regulated, 22 up-regulated), 62 between KL1 and TL3 (37 down-regulated, 25 up-regulated), 40 between TL1 and TL2 (16 down-regulated, 24 up-regulated), 34 between TL1 and TL3 (8 down-regulated, 26 up-regulated), and 40 between TL2 and TL3 (9 down-regulated, 31 up-regulated). Compared with tartary buckwheat, most of the flavonoid metabolites of common buckwheat were down-regulated. However, in common buckwheat, most of the flavonoid metabolites in leaves were up-regulated as the color of the flower changed from white to red. After taking intersection of each comparison group in a Venn diagram (Figure 5A), 44 common differential metabolites were observed among comparison groups KL1 vs TL1, KL1 vs TL2, and KL1 vs TL3. However, only 8 common differential metabolites were shown amongst comparison groups TL1 vs TL2, TL1 vs TL3, and TL2 vs TL3. The results showed that the flavonoid metabolites that caused the difference between KL1 and TL1, TL2, TL3 were essentially identical.

The differential flavonoid metabolites from each comparison group was annotated by the Kyoto Encyclopedia of Genes and Genomes (KEGG) database (Supplementary Table S3). The KEGG classification results indicated that the differential flavonoid metabolites of comparison groups were involved in anthocyanin biosynthesis, flavone and flavonol biosynthesis, isoflavonoid biosynthesis, biosynthesis of secondary metabolites, flavonoid biosynthesis, biosynthesis of phenylpropanoids, and metabolic pathways.

## 3. Discussion

Buckwheat food is becoming increasingly popular with consumers because of its many health benefits. At present, the use of buckwheat is mainly limited to its grains, which are made into a variety of foods and the buckwheat tea that is widely enjoyed by Asian and European countries [17,18,24]. Leaf tea, such as green tea, sea buckthorn leaf tea, jujube leaf tea, and peppermint leaf tea, is extremely popular because its constituent flavonoids have positive effects on health [25,26,27,28]. Buckwheat leaves are also rich in flavonoids, but the utilization is extremely inadequate. Thus, this study aimed to provide a theoretical basis for the utilization of buckwheat leaves. The total flavonoid content of red-flowered common buckwheat leaves was the highest, followed by tartary buckwheat, which was consistent with the results of a previous research on buckwheat flowers (unpublished). The enrichment analysis of the differential metabolites revealed that differential metabolites were mainly involved in anthocyanin, isoflavonoid, flavone, and flavonol biosynthesis (Figure 5B–D; Supplementary Table S3).

### 3.1. Differential Metabolites Involved in Anthocyanin Biosynthesis

Comparison groups showed that the pelargonin was only present in common buckwheat leaves and absent in those of tartary buckwheat. Noda et al. [29] reported that the red and magenta flowers contain pelargonidin-based anthocyanins, whereas the blue-hued flowers contain delphinidin-based anthocyanins. In the present study, two delphinidin-based anthocyanins (i.e., tulipanin and mirtillin) were only detected in tartary buckwheat leaves. The cyanidin 3-*O*-rutinoside was the major component of anthocyanins, which mainly contribute to the red color of tartary buckwheat sprouts [30,31]. The result of this study also showed that the cyanidin 3-*O*-rutinoside content in all samples was the highest compared with other anthocyanins, but the differences amongst the samples were insignificant.

### 3.2. Differential Metabolites Involved in Isoflavonoid Biosynthesis

Isoflavones are generally exclusively present in legumes such as soybeans, and play important roles in plant defense and nodules [32]. In the present study, seven isoflavones were detected in buckwheat leaves, of which the sissotrin was only detected in common buckwheat leaves, whereas the 6-hydroxydaidzein was only detected in tartary buckwheat leaves. Sissotrin belongs to biochanin A, which is an isoflavone that is commonly found in white clover and red clover and has antifungal activity, selective inhibitory effects on bacteria, and a stimulating activity on soil microorganisms [33]. However, sissotrin can impair glucose tolerance and does not have antihyperglycemic activity [34]. The 6-Hydroxydaidzein has almost the same activity as daidzein, which has DPPH radical scavenging activity but shows low antimutagenic activity [35].

### 3.3. Differential Metabolites Involved in Flavone and Flavonol Biosynthesis

Compared with tartary buckwheat, most of the flavonol metabolites in the leaves of common buckwheat were down-regulated. Syringetin induces human osteoblast differentiation [36] and can possibly treat cancer [37]. In the present study, the syringetin content in common buckwheat leaves was down-regulated compared with that tartary buckwheat. Meanwhile, the syringetin derivative, that is syringetin 3-*O*-hexoside, was only detected in TL2 and TL3. Kaempferide has a hypolipidemic effect on hyperlipidemia caused by high-fat diet [38] which was only detected in tartary buckwheat leaves. Quercetin 3-*O*-rutinoside (rutin) is a citrus flavonoid that has many potential health benefits, such as strengthening blood vessels, aiding the usage of vitamin C, and is involved in collagen production. It can also reduce cholesterol levels, decrease blood clots, and lower blood pressure [7,39,40,41,42]. In the present study, rutin was detected in all samples and the highest amongst flavonol metabolites. However, the differences amongst samples were insignificant.

Most of the flavone metabolites were down-regulated in common buckwheat leaves compared with tartary buckwheat, whereas the flavone C-glycosides metabolites were mostly up-regulated in common buckwheat. This result was consistent with the PCA conclusion that flavone C-glycosides remarkably contributed to common buckwheat leaves, but flavone had considerable contribution to tartary buckwheat. Metabolites screening results showed that all luteolin derivatives were up-regulated in the common buckwheat leaves compared with tartary buckwheat, and apigenin C-glucoside was also up-regulated in common buckwheat leaves. The result was similar to the conclusion of a previous study in cotyledons that common buckwheat sprouts are rich in flavone C-glycosides of luteolin and apigenin compared with tartary buckwheat [2]. Luteolin showed strong DPPH scavenging activity, and apigenin can inhibit UV-induced skin tumorigenesis [22]. Tricin is a methylated flavone that is widely distributed in the bran and hull of cereals [43]. Tricin has considerable potential as a functional agent for glycemic control and also had anti-inflammatory and anti-cancer effects [44,45,46]. In this study, tricin and its derivatives were only detected in tartary buckwheat except tricin 7-*O*-hexoside. The natural flavone acacetin can provide remarkable cardioprotection [47] and induce apoptosis in cancer cell lines through the inhibition of different enzymes systems [48]. Acacetin also possesses antimutagenic, antiplasmodial, antiperoxidant, anti-inflammatory, and anticancer effects [49]. In the present study, acacetin was detected in both tartary buckwheat and common buckwheat leaves, but its content was higher in the former than the latter.

In conclusion, buckwheat leaves were rich in flavonoids. The flavonoids content of the leaves from red-flowered common buckwheat and tartary buckwheat was higher than the other samples. A total of 182 flavonoid metabolites were detected by UPLC-ESI-MS/MS. Flavonoid metabolites varied widely in different samples, and both tartary buckwheat and common buckwheat leaves had their own unique metabolites. Hence, the tartary buckwheat and red-flowered common buckwheat leaves have considerable potential for development and utilization in the future, such as in buckwheat leaf tea.

## 4. Materials and Methods

### 4.1. Plant Materials

One tartary buckwheat variety and three common buckwheat varieties were selected as experimental varieties. These four buckwheat varieties have excellent quality characteristics and are important buckwheat cultivars in Northwest China. The difference in appearance among the varieties was mainly reflected in the difference of flower colors, which were green, white, pink and red. The seeds used in the experiment were provided by the Minor grain research group of the Agronomy College, Northwest A & F University. The experiment was carried out in the north campus experimental field of Northwest A & F University, Yangling, China (108°24′ E, 34°20′ N, altitude 521 m). A randomized field plot was designed. All buckwheat varieties were sown on June 15, 2017, as in production. At the flowering stage (the tartary buckwheat was 50 days after sowing and the common buckwheat was 40 days after sowing), the leaves of four buckwheat varieties were collected. Samples were frozen in liquid nitrogen immediately after collection and stored at −80 °C.

### 4.2. Determination of Total Flavonoids Content

Approximately 3.0 g of leaves from each variety were freeze-dried by using a vacuum freeze dryer (LGJ-10, Songyuanhuaxing Technology Develop Co., Ltd., Beijing, China) and then ground into powder using a mortar. The total flavonoid content of 0.02 g leaves was determined in accordance with the protocol of Plant Flavonoids Test kit (Suzhou Comin Biotechnology Co., Ltd., Suzhou, China).

### 4.3. Sample Preparation and Extraction for Metabolomic Analysis

The preparation and extraction of the samples was followed the method of Dong et al. [19]. The freeze-dried leaves were ground into powder and extracted overnight with 70% aqueous methanol. The dissolved sample was stored in refrigerator at 4 °C overnight and centrifuged at 10,000 *g* for 10 min. The extracts were absorbed (CNWBOND Carbon-GCB SPE Cartridge, 250 mg, 3 mL; ANPEL, Shanghai, China) and filtrated (SCAA-104, 0.22 μm pore size; ANPEL, Shanghai, China) before LC-MS analysis.

### 4.4. Ultra Performance Liquid Chromatography (UPLC) Conditions

The sample extracts were analyzed in accordance with the method of Wang et al. [8] by using an UPLC-ESI-MS/MS system (UPLC, Shim-pack UFLC SHIMADZU CBM30A system; MS, Applied Biosystems 6500 Q TRAP). Detailed UPLC analytical conditions are shown in Table 1. After UPLC, the effluent was alternatively connected to an ESI-triple quadrupole-linear ion trap (Q TRAP)-MS.

### 4.5. ESI-Q TRAP-MS/MS

Mass spectrometry followed the method of Chen et al. [50]. Linear ion trap (LIT) and triple quadrupole (QQQ) scans were acquired on a triple quadrupole–linear ion trap mass spectrometer (Q TRAP), API 6500 Q TRAP LC/MS/MS System, equipped with an ESI Turbo Ion-Spray interface, which was operated in both positive and negative ion mode and controlled via Analyst 1.6 (AB Sciex). The ESI source operation parameters were as follows: ion source, turbo spray; source temperature 500 °C; ion spray voltage (IS) 5500 V; ion source gas I (GSI), gas II(GSII) and curtain gas (CUR) were set at 55, 60, and 25.0 psi, respectively. The collision gas (CAD) was high. Instrument tuning and mass calibration were performed with 10 and 100 μmol/L polypropylene glycol solutions in QQQ and LIT modes, respectively. QQQ scans were acquired as multiple reaction monitoring (MRM) experiments with collision gas (nitrogen) set to 5 psi. Declustering potential (DP) and collision energy (CE) for individual MRM transitions were done with further DP and CE optimization. A specific set of MRM transitions was monitored for each period according to the metabolites eluted within this period.

### 4.6. Qualitative and Quantitative Analysis of Metabolites

Qualitative and quantitative analyses of metabolites followed the methods of Wang [8] and Fraga [51]. Based on the self-built database MWDB (Metware Biotechnology Co., Ltd. Wuhan, China) and the public database of metabolite information, the qualitative analysis of the primary and secondary spectral data of mass spectrometry was performed. The analysis removed the isotope signal, a repetitive signal containing K^+^ ions, Na^+^ ions, NH_4_^+^ ions, and a repetitive signal of fragment ions with larger molecular weight themselves. The quantitative analysis of metabolites was performed using MRM analysis of QQQ mass spectrometry. After obtaining metabolite mass spectrometry data of different samples, peak area integration was performed on the mass spectrum peaks of all the substances and the mass spectrum peaks of the same metabolite in different samples were integrated for correction.

### 4.7. Sample Quality Control Analysis

**Figure 6:**
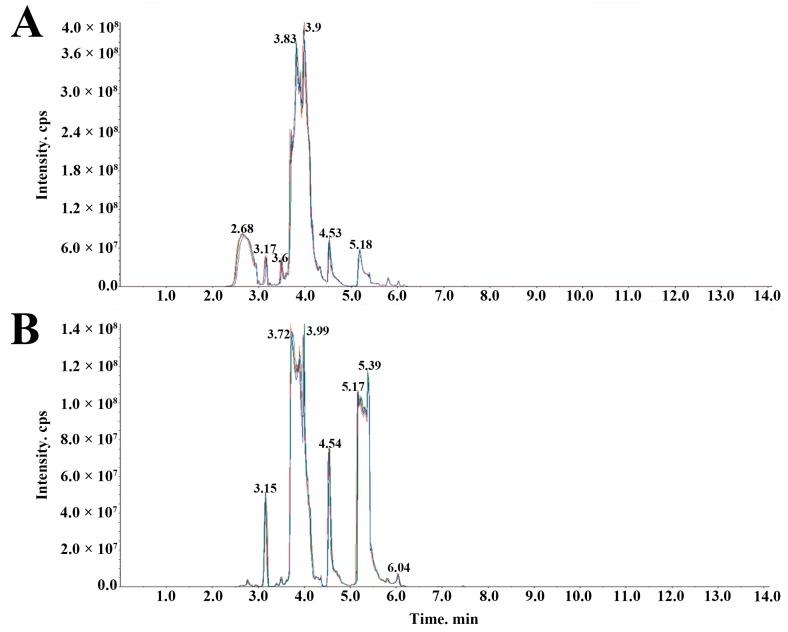
The stacking diagram of total ions current (TIC) maps from quality control samples (QC) mass spectrometry. (**A**) TIC of positive ion multiple reaction monitoring (MRM). (**B**) TIC of negative ion MRM.

The high stability of the instrument guaranteed data repeatability and reliability. To check the repeatability of metabolite extraction and detection, overlapping display analysis of total ions current (TIC) maps for mass spectrometry analysis of different quality control samples (QC) were drawn. The QC was prepared by mixing sample extracts, and for every 10 test samples one QC was inserted. The stacking diagram of TIC maps from QC mass spectrometry are shown in Figure 6. The TIC curve of the metabolites showed high overlap, that was, the retention time and peak intensity were consistent, thus indicating that the signal stability was good when the mass spectrometer detected the same sample at different times.

### 4.8. Statistical Analysis

Three biological replicates were performed in each experiment. Statistical analysis was performed by using Microsoft Office Excel 2016 and SPSS 23.0 (IBM Corporation, Armonk, NY, USA). Comparisons were statistically evaluated by using one-way analysis of variance (ANOVA) and the Duncan’s multiple range test to determine the significant difference. Significance was set at *p* < 0.05. The figures used in this article were drawn by using OriginPro 2016 (OriginLab Corporation, Northampton, MA, USA) coupled with Adobe Illustrator CC. Hierarchical clustering analysis (HCA), PCA and OPLS-DA were carried out by using R (http://www.r-project.org/) in accordance with previously described methods [52].

## Figures and Tables

**Table 1:** Analytical conditions of the Ultra Performance Liquid Chromatography (UPLC).

Conditions	Parameters	
Column	Waters ACQUITY UPLC HSS T3 C18 (1.8 µm, 2.1 mm × 100 mm)	
Solvent system	Mobile phase A (0.04% acetic acid in water) Mobile phase B (0.04% acetic acid in acetonitrile)	
Gradient program	0 min	95:5 *v*/*v* (Mobile phase A: Mobile phase B)
	11.0 min	5:95 *v*/*v*
	12.0 min	5:95 *v*/*v*
	12.1 min	95:5 *v*/*v*
	15.0 min	95:5 *v*/*v*
Flow rate	0.40 mL/min	
Column temperature	40 °C	
Injection volume	2 μL	

## Supplementary Materials

The following are available online at https://www.mdpi.com/1420-3049/24/7/1310/s1, Supplementary Table S1: A list of the 182 metabolites detected in this study; Supplementary Table S2: The screening results of differential flavonoid metabolites; Supplementary Table S3: KEGG annotated results of differential flavonoid metabolites.
