# Supplementary material for: Analysis of Flavonoid Metabolites in Buckwheat Leaves Using UPLC-ESI-MS/MS
Source: Molecules. 2019 Apr 3;24(7):1310. doi: 10.3390/molecules24071310 (PMC6479795; doi:10.3390/molecules24071310)
Supplement: Supplementary file 1 [file molecules-24-01310-s001.zip › Supplementary Table S1.docx]

**Supplementary Table 1** A list of the 182 metabolites detected in this study.

| **Index** | **Q1 (Da)** | **Q3 (Da)** | **Rt (min)** | **Molecular Weight (Da)** | **Ionization model** | **KEGG ID** | **Compounds** | **Class** |
| --- | --- | --- | --- | --- | --- | --- | --- | --- |
| pme1777 | 6.11E+02 | 2.88E+02 | 2.15E+00 | 6.11E+02 | Protonated | C08639 | "Cyanidin 3,5-O-diglucoside (Cyanin)" | Anthocyanins |
| pmb0541 | 6.97E+02 | 6.97E+02 | 2.24E+00 | 6.97E+02 | Protonated | - | Cyanidin 3-O-glucosyl-malonylglucoside | Anthocyanins |
| pme3256 | 6.11E+02 | 3.03E+02 | 2.37E+00 | 6.11E+02 | Protonated | C16315 | Delphinidin 3-O-rutinoside (Tulipanin) | Anthocyanins |
| pme1398 | 4.65E+02 | 3.03E+02 | 2.38E+00 | 4.65E+02 | Protonated | C12138 | Delphinidin 3-O-glucoside (Mirtillin) | Anthocyanins |
| pme1793 | 5.95E+02 | 2.72E+02 | 2.40E+00 | 5.95E+02 | Protonated | C08725 | Pelargonin | Anthocyanins |
| pme1786 | 6.55E+02 | 3.31E+02 | 2.45E+00 | 6.55E+02 | Protonated | C08718 | "Malvidin 3,5-diglucoside (Malvin)" | Anthocyanins |
| pme1773 | 5.95E+02 | 2.88E+02 | 2.62E+00 | 5.95E+02 | Protonated | C08620 | Cyanidin 3-O-rutinoside (Keracyanin) | Anthocyanins |
| pme3391 | 4.79E+02 | 3.17E+02 | 2.63E+00 | 4.79E+02 | Protonated | C12139 | Petunidin 3-O-glucoside | Anthocyanins |
| pme3392 | 4.33E+02 | 2.71E+02 | 2.75E+00 | 4.33E+02 | Protonated | - | Pelargonidin 3-O-beta-D-glucoside（Callistephin chloride) | Anthocyanins |
| pme0443 | 4.93E+02 | 3.32E+02 | 2.87E+00 | 4.93E+02 | Protonated | - | Malvidin 3-O-galactoside | Anthocyanins |
| pme0444 | 4.93E+02 | 3.32E+02 | 2.87E+00 | 4.93E+02 | Protonated | C12140 | Malvidin 3-O-glucoside (Oenin) | Anthocyanins |
| pma1590 | 4.63E+02 | 3.01E+02 | 3.00E+00 | 4.63E+02 | Protonated | - | Peonidin O-hexoside | Anthocyanins |
| pmb0545 | 4.77E+02 | 3.16E+02 | 3.31E+00 | 4.77E+02 | Protonated | - | Rosinidin O-hexoside | Anthocyanins |
| pme3609 | 2.87E+02 | 2.32E+02 | 3.45E+00 | 2.87E+02 | Protonated | C05905 | Cyanidin | Anthocyanins |
| pmb0563 | 3.01E+02 | 2.74E+02 | 3.98E+00 | 3.01E+02 | Protonated | C08726 | Peonidin | Anthocyanins |
| pmb2957 | 4.65E+02 | 2.85E+02 | 2.50E+00 | 4.66E+02 | [M-H]- | - | Cyanidin O-syringic acid | Anthocyanins |
| pme0094 | 4.47E+02 | 2.84E+02 | 2.55E+00 | 4.48E+02 | [M-H]- | C08604 | Cyanidin 3-O-glucoside (Kuromanin) | Anthocyanins |
| pmb2961 | 5.47E+02 | 5.03E+02 | 2.87E+00 | 5.48E+02 | [M-H]- | - | Peonidin O-malonylhexoside | Anthocyanins |
| pme3285 | 2.75E+02 | 1.39E+02 | 3.28E+00 | 2.74E+02 | [M+H]+ | C09320 | "Afzelechin (3,5,7,4'-Tetrahydroxyflavan)" | Flavanone |
| pme2950 | 6.11E+02 | 3.03E+02 | 4.00E+00 | 6.10E+02 | [M+H]+ | C09755 | Hesperetin 7-rutinoside (Hesperidin) | Flavanone |
| pme0002 | 6.11E+02 | 3.03E+02 | 4.04E+00 | 6.10E+02 | [M+H]+ | C09806 | Hesperetin 7-O-neohesperidoside (Neohesperidin) | Flavanone |
| pme0372 | 4.35E+02 | 2.73E+02 | 4.17E+00 | 4.34E+02 | [M+H]+ | C09099 | Naringenin 7-O-glucoside (Prunin) | Flavanone |
| pma0791 | 5.21E+02 | 3.18E+02 | 4.46E+00 | 5.20E+02 | [M+H]+ | - | Naringenin O-malonylhexoside | Flavanone |
| pmb0686 | 5.37E+02 | 2.90E+02 | 4.48E+00 | 5.36E+02 | [M+H]+ | - | Eriodictyol O-malonylhexoside | Flavanone |
| pme0377 | 2.73E+02 | 1.53E+02 | 5.60E+00 | 2.72E+02 | [M+H]+ | C00509 | Naringenin | Flavanone |
| pme3217 | 2.57E+02 | 1.37E+02 | 6.05E+00 | 2.56E+02 | [M+H]+ | C08650 | Isoliquiritigenin | Flavanone |
| pme1399 | 3.55E+02 | 1.79E+02 | 8.24E+00 | 3.54E+02 | [M+H]+ | C16417 | Xanthohumol | Flavanone |
| pmb2970 | 6.25E+02 | 4.63E+02 | 2.29E+00 | 6.26E+02 | [M-H]- | - | Hesperetin O-hexosyl-O-hexoside | Flavanone |
| pme1598 | 4.63E+02 | 3.01E+02 | 3.81E+00 | 4.64E+02 | [M-H]- | - | Hesperetin 5-O-glucoside | Flavanone |
| pme0330 | 5.79E+02 | 1.35E+02 | 3.97E+00 | 5.80E+02 | [M-H]- | C09789 | Naringenin 7-O-neohesperidoside (Naringin) | Flavanone |
| pmb2979 | 5.49E+02 | 3.87E+02 | 3.99E+00 | 5.50E+02 | [M-H]- | - | Hesperetin O-malonylhexoside | Flavanone |
| pme1580 | 2.87E+02 | 1.35E+02 | 5.03E+00 | 2.88E+02 | [M-H]- | C05631 | Eriodictyol | Flavanone |
| pme2984 | 5.93E+02 | 2.85E+02 | 4.90E+00 | 5.94E+02 | [M-H]- | C09830 | Isosakuranetin-7-neohesperidoside (Poncirin) | Flavanone |
| pme2957 | 2.71E+02 | 1.51E+02 | 5.45E+00 | 2.72E+02 | [M-H]- | C06561 | Naringenin chalcone | Flavanone |
| pme3439 | 2.71E+02 | 1.35E+02 | 5.30E+00 | 2.72E+02 | [M-H]- | C08578 | Butein | Flavanone |
| pme1201 | 2.73E+02 | 1.67E+02 | 5.52E+00 | 2.74E+02 | [M-H]- | C00774 | Phloretin | Flavanone |
| pme3461 | 3.01E+02 | 1.51E+02 | 5.71E+00 | 3.02E+02 | [M-H]- | C09756 | Homoeriodictyol | Flavanone |
| pme2319 | 3.01E+02 | 2.42E+02 | 5.63E+00 | 3.02E+02 | [M-H]- | C01709 | Hesperetin | Flavanone |
| pme1599 | 3.01E+02 | 1.35E+02 | 6.07E+00 | 3.02E+02 | [M-H]- | - | 7-O-Methyleriodictyol | Flavanone |
| pmc1990 | 2.99E+02 | 7.48E+01 | 6.74E+00 | 3.00E+02 | [M-H]- | - | "4'-Hydroxy-5,7-dimethoxyflavanone" | Flavanone |
| pme3464 | 2.85E+02 | 1.64E+02 | 6.81E+00 | 2.86E+02 | [M-H]- | C05334 | Isosakuranetin (4'-Methylnaringenin) | Flavanone |
| pme2979 | 2.55E+02 | 1.51E+02 | 7.03E+00 | 2.56E+02 | [M-H]- | C09827 | Pinocembrin (Dihydrochrysin) | Flavanone |
| pmb0587 | 6.39E+02 | 6.40E+02 | 3.27E+00 | 6.38E+02 | [M+H]+ | - | Chrysoeriol O-glucuronic acid-O-hexoside | Flavone |
| pmb0588 | 6.11E+02 | 4.49E+02 | 3.38E+00 | 6.10E+02 | [M+H]+ | - | "Luteolin 3',7-di-O-glucoside" | Flavone |
| pma6499 | 5.09E+02 | 3.48E+02 | 3.46E+00 | 5.08E+02 | [M+H]+ | - | Limocitrin O-hexoside | Flavone |
| pma0249 | 4.79E+02 | 3.03E+02 | 3.54E+00 | 4.78E+02 | [M+H]+ | - | Selgin 5-O-hexoside | Flavone |
| pmb0732 | 6.69E+02 | 6.69E+02 | 3.71E+00 | 6.68E+02 | [M+H]+ | - | Tricin 5-O-feruloylhexoside | Flavone |
| pmb0590 | 4.61E+02 | 2.57E+02 | 3.75E+00 | 4.60E+02 | [M+H]+ | - | Acacetin O-glucuronic acid | Flavone |
| pme0359 | 4.33E+02 | 3.13E+02 | 3.75E+00 | 4.32E+02 | [M+H]+ | - | Apigenin 5-O-glucoside | Flavone |
| pme2459 | 4.49E+02 | 2.87E+02 | 3.77E+00 | 4.48E+02 | [M+H]+ | C03951 | Luteolin 7-O-glucoside (Cynaroside) | Flavone |
| pmb0569 | 5.09E+02 | 3.48E+02 | 4.01E+00 | 5.08E+02 | [M+H]+ | - | Syringetin 5-O-hexoside | Flavone |
| pme0333 | 5.79E+02 | 2.71E+02 | 4.02E+00 | 5.78E+02 | [M+H]+ | C12627 | Apigenin 7-O-neohesperidoside (Rhoifolin) | Flavone |
| pmb0733 | 6.39E+02 | 3.32E+02 | 4.02E+00 | 6.38E+02 | [M+H]+ | - | Tricin 5-O-rutinoside | Flavone |
| pmb0600 | 6.09E+02 | 3.02E+02 | 4.03E+00 | 6.08E+02 | [M+H]+ | - | Chrysoeriol 7-O-rutinoside | Flavone |
| pmb0601 | 5.95E+02 | 3.02E+02 | 4.05E+00 | 5.94E+02 | [M+H]+ | - | Chrysoeriol O-hexosyl-O-pentoside | Flavone |
| pmb0602 | 5.09E+02 | 2.84E+02 | 4.10E+00 | 5.08E+02 | [M+H]+ | - | Syringetin 7-O-hexoside | Flavone |
| pmb0603 | 6.25E+02 | 3.32E+02 | 4.12E+00 | 6.24E+02 | [M+H]+ | - | Chrysoeriol O-hexosyl-O-hexoside | Flavone |
| pmb0605 | 4.33E+02 | 2.72E+02 | 4.15E+00 | 4.32E+02 | [M+H]+ | C04608 | Apigenin 7-O-glucoside (Cosmosiin) | Flavone |
| pmb0736 | 4.93E+02 | 3.32E+02 | 4.24E+00 | 4.92E+02 | [M+H]+ | - | Tricin 7-O-hexoside | Flavone |
| pmb0607 | 4.63E+02 | 2.88E+02 | 4.25E+00 | 4.62E+02 | [M+H]+ | - | Chrysoeriol 7-O-hexoside | Flavone |
| pma0760 | 5.65E+02 | 3.18E+02 | 4.32E+00 | 5.64E+02 | [M+H]+ | - | Selgin O-malonylhexoside | Flavone |
| pma0795 | 5.51E+02 | 3.47E+02 | 4.52E+00 | 5.50E+02 | [M+H]+ | - | Tricetin O-malonylhexoside | Flavone |
| pmb0578 | 6.55E+02 | 2.88E+02 | 4.59E+00 | 6.54E+02 | [M+H]+ | - | Luteolin O-sinapoylhexoside | Flavone |
| pmb0579 | 6.69E+02 | 3.02E+02 | 4.82E+00 | 6.68E+02 | [M+H]+ | - | Chrysoeriol O-sinapoylhexoside | Flavone |
| pmb0580 | 4.17E+02 | 2.56E+02 | 4.95E+00 | 4.16E+02 | [M+H]+ | - | Chrysin 5-O-glucoside (Toringin) | Flavone |
| pma6199 | 4.17E+02 | 2.56E+02 | 4.96E+00 | 4.16E+02 | [M+H]+ | - | Chrysin O-hexoside | Flavone |
| pme0089 | 2.87E+02 | 2.87E+02 | 5.00E+00 | 2.86E+02 | [M+H]+ | C01514 | Luteolin | Flavone |
| pmb0725 | 6.69E+02 | 6.69E+02 | 5.04E+00 | 6.68E+02 | [M+H]+ | - | Tricin 7-O-feruloylhexoside | Flavone |
| pma0253 | 4.77E+02 | 3.16E+02 | 5.27E+00 | 4.76E+02 | [M+H]+ | - | O-methylChrysoeriol 5-O-hexoside | Flavone |
| pma6638 | 4.77E+02 | 3.28E+02 | 5.27E+00 | 4.76E+02 | [M+H]+ | - | O-methylChrysoeriol 7-O-hexoside | Flavone |
| pme0379 | 2.71E+02 | 2.15E+02 | 5.62E+00 | 2.70E+02 | [M+H]+ | C01477 | Apigenin | Flavone |
| pme1662 | 2.87E+02 | 2.87E+02 | 6.92E+00 | 2.86E+02 | [M+H]+ | C09833 | sakuranetin | Flavone |
| pme1518 | 4.03E+02 | 3.73E+02 | 7.02E+00 | 4.02E+02 | [M+H]+ | C10112 | Nobiletin | Flavone |
| pme1550 | 3.73E+02 | 3.73E+02 | 7.50E+00 | 3.72E+02 | [M+H]+ | C10190 | Tangeretin | Flavone |
| pmb2987 | 4.87E+02 | 2.83E+02 | 2.57E+00 | 4.88E+02 | [M-H]- | - | Acacetin O-acetyl hexoside | Flavone |
| pmb2991 | 7.39E+02 | 2.69E+02 | 3.72E+00 | 7.40E+02 | [M-H]- | - | Apigenin O-hexosyl-O-rutinoside | Flavone |
| pmb3028 | 5.35E+02 | 3.29E+02 | 3.68E+00 | 5.36E+02 | [M-H]- | - | Tricin O-sinapic acid | Flavone |
| pmb3041 | 5.21E+02 | 3.29E+02 | 3.74E+00 | 5.22E+02 | [M-H]- | - | Tricin O-saccharic acid | Flavone |
| pme0367 | 5.77E+02 | 2.69E+02 | 3.82E+00 | 5.78E+02 | [M-H]- | - | Apigenin 7-rutinoside (Isorhoifolin) | Flavone |
| pmb2997 | 7.99E+02 | 4.61E+02 | 3.88E+00 | 8.00E+02 | [M-H]- | - | Chrysoeriol O-hexosyl-O-hexosyl-O-Glucuronic acid | Flavone |
| pmb2978 | 5.63E+02 | 2.69E+02 | 3.93E+00 | 5.64E+02 | [M-H]- | - | Apigenin O-hexosyl-O-pentoside | Flavone |
| pmb2999 | 4.61E+02 | 2.85E+02 | 3.80E+00 | 4.62E+02 | [M-H]- | - | Chrysoeriol 5-O-hexoside | Flavone |
| pmb3042 | 4.91E+02 | 3.29E+02 | 3.94E+00 | 4.92E+02 | [M-H]- | - | Tricin 5-O-hexoside | Flavone |
| pmb3045 | 5.05E+02 | 3.30E+02 | 4.14E+00 | 5.06E+02 | [M-H]- | - | Tricin O-glucuronic acid | Flavone |
| pmb3007 | 4.75E+02 | 2.85E+02 | 4.15E+00 | 4.76E+02 | [M-H]- | - | Chrysoeriol O-glucuronic acid | Flavone |
| pme3300 | 3.01E+02 | 1.49E+02 | 4.38E+00 | 3.02E+02 | [M-H]- | C10192 | Tricetin | Flavone |
| pmb3014 | 6.41E+02 | 2.85E+02 | 4.50E+00 | 6.42E+02 | [M-H]- | - | Luteolin O-eudesmic acid-O-hexoside | Flavone |
| pme3473 | 2.71E+02 | 1.51E+02 | 5.59E+00 | 2.72E+02 | [M-H]- | C09614 | Butin | Flavone |
| pme0363 | 2.99E+02 | 2.84E+02 | 5.71E+00 | 3.00E+02 | [M-H]- | C04293 | Chrysoeriol | Flavone |
| pmb2850 | 3.29E+02 | 3.14E+02 | 5.68E+00 | 3.30E+02 | [M-H]- | - | Tricin | Flavone |
| pmb3037 | 5.71E+02 | 3.30E+02 | 5.67E+00 | 5.72E+02 | [M-H]- | - | Tricin O-malonyl shikimic acid | Flavone |
| pmb3039 | 7.03E+02 | 3.29E+02 | 5.94E+00 | 7.04E+02 | [M-H]- | - | Tricin O-oxalic acid O-coumaroyl shikimic acid | Flavone |
| pmb3053 | 5.23E+02 | 3.29E+02 | 5.95E+00 | 5.24E+02 | [M-H]- | - | Tricin O-eudesmic acid | Flavone |
| pme1541 | 2.83E+02 | 2.68E+02 | 6.98E+00 | 2.84E+02 | [M-H]- | C01470 | Acacetin | Flavone |
| pma6558 | 3.13E+02 | 2.98E+02 | 7.23E+00 | 3.14E+02 | [M-H]- | - | Velutin | Flavone |
| pmb0619 | 7.75E+02 | 7.75E+02 | 2.58E+00 | 7.74E+02 | [M+H]+ | - | Eriodictiol 6-C-hexoside 8-C-hexoside-O-hexoside | Flavone C-glycosides |
| pmb0615 | 7.89E+02 | 7.89E+02 | 2.63E+00 | 7.88E+02 | [M+H]+ | - | Hesperetin C-hexosyl-O-hexosyl-O-hexoside | Flavone C-glycosides |
| pmb0618 | 6.27E+02 | 4.30E+02 | 2.76E+00 | 6.26E+02 | [M+H]+ | - | 8-C-hexosyl-hesperetin O-hexoside | Flavone C-glycosides |
| pmb0620 | 7.87E+02 | 3.44E+02 | 2.78E+00 | 7.86E+02 | [M+H]+ | - | Chrysoeriol 6-C-hexoside 8-C-hexoside-O-hexoside | Flavone C-glycosides |
| pmb0622 | 6.11E+02 | 4.12E+02 | 2.89E+00 | 6.10E+02 | [M+H]+ | - | C-hexosyl-luteolin O-hexoside | Flavone C-glycosides |
| pmb0621 | 6.41E+02 | 6.23E+02 | 2.90E+00 | 6.40E+02 | [M+H]+ | - | C-hexosyl-isorhamnetin O-hexoside | Flavone C-glycosides |
| pmb0623 | 6.25E+02 | 3.98E+02 | 2.95E+00 | 6.24E+02 | [M+H]+ | - | 6-C-hexosyl chrysoeriol O-hexoside | Flavone C-glycosides |
| pmb0624 | 6.11E+02 | 3.29E+02 | 3.05E+00 | 6.10E+02 | [M+H]+ | - | 6-C-hexosyl-luteolin O-hexoside | Flavone C-glycosides |
| pmb0639 | 7.57E+02 | 7.57E+02 | 3.05E+00 | 7.56E+02 | [M+H]+ | - | 8-C-hexosyl-apigenin O-hexosyl-O-hexoside | Flavone C-glycosides |
| pmb0691 | 7.57E+02 | 7.57E+02 | 3.06E+00 | 7.56E+02 | [M+H]+ | - | Luteolin C-hexosyl-O-rhamnoside O-hexoside | Flavone C-glycosides |
| pmb0629 | 4.63E+02 | 3.80E+02 | 3.06E+00 | 4.62E+02 | [M+H]+ | - | Chrysoeriol 6-C-hexoside | Flavone C-glycosides |
| pma6371 | 6.11E+02 | 5.76E+02 | 3.27E+00 | 6.10E+02 | [M+H]+ | - | "di-C,C-hexosyl-luteolin" | Flavone C-glycosides |
| pmb0645 | 6.27E+02 | 4.47E+02 | 3.37E+00 | 6.26E+02 | [M+H]+ | - | 6-C-hexosyl-hesperetin O-hexoside | Flavone C-glycosides |
| pmb0647 | 5.81E+02 | 4.49E+02 | 3.38E+00 | 5.80E+02 | [M+H]+ | - | 8-C-hexosyl-luteolin O-pentoside | Flavone C-glycosides |
| pmb0696 | 6.25E+02 | 3.98E+02 | 3.41E+00 | 6.24E+02 | [M+H]+ | - | 8-C-hexosyl chrysoeriol O-hexoside | Flavone C-glycosides |
| pma6496 | 4.49E+02 | 3.00E+02 | 3.47E+00 | 4.48E+02 | [M+H]+ | - | Luteolin 6-C-glucoside | Flavone C-glycosides |
| pmb0652 | 5.65E+02 | 3.98E+02 | 3.55E+00 | 5.64E+02 | [M+H]+ | - | C-hexosyl-apigenin O-pentoside | Flavone C-glycosides |
| pma6254 | 7.57E+02 | 7.57E+02 | 3.61E+00 | 7.56E+02 | [M+H]+ | - | C-hexosyl-apigenin O-caffeoylhexoside | Flavone C-glycosides |
| pmb0665 | 6.11E+02 | 3.00E+02 | 3.67E+00 | 6.10E+02 | [M+H]+ | - | Luteolin 8-C-hexosyl-O-hexoside | Flavone C-glycosides |
| pma1108 | 4.33E+02 | 2.72E+02 | 3.73E+00 | 4.32E+02 | [M+H]+ | - | Apigenin C-glucoside | Flavone C-glycosides |
| pme1624 | 4.33E+02 | 3.13E+02 | 3.74E+00 | 4.32E+02 | [M+H]+ | C01714 | Isovitexin | Flavone C-glycosides |
| pma0724 | 4.35E+02 | 3.40E+02 | 3.74E+00 | 4.34E+02 | [M+H]+ | - | Naringenin C-hexoside | Flavone C-glycosides |
| pmb0661 | 6.09E+02 | 4.63E+02 | 3.78E+00 | 6.08E+02 | [M+H]+ | - | Chrysoeriol C-hexosyl-O-rhamnoside | Flavone C-glycosides |
| pma6218 | 4.19E+02 | 3.84E+02 | 3.81E+00 | 4.18E+02 | [M+H]+ | - | O-methylnaringenin C-pentoside | Flavone C-glycosides |
| pmb0701 | 4.63E+02 | 3.68E+02 | 3.85E+00 | 4.62E+02 | [M+H]+ | - | Chrysoeriol 8-C-hexoside | Flavone C-glycosides |
| pmb0680 | 7.41E+02 | 7.41E+02 | 3.94E+00 | 7.40E+02 | [M+H]+ | - | C-hexosyl-apigenin O-p-coumaroylhexoside | Flavone C-glycosides |
| pmb0673 | 4.03E+02 | 3.68E+02 | 3.99E+00 | 4.02E+02 | [M+H]+ | - | Apigenin 6-C-pentoside | Flavone C-glycosides |
| pma6515 | 7.55E+02 | 3.32E+02 | 4.58E+00 | 7.54E+02 | [M+H]+ | - | C-hexosyl-chrysin O-feruloylhexoside | Flavone C-glycosides |
| pmb3023 | 4.49E+02 | 3.29E+02 | 3.30E+00 | 4.50E+02 | [M-H]- | - | Eriodictyol C-hexoside | Flavone C-glycosides |
| pmb3024 | 4.47E+02 | 3.27E+02 | 3.37E+00 | 4.48E+02 | [M-H]- | - | Luteolin C-hexoside | Flavone C-glycosides |
| pme3224 | 5.77E+02 | 4.13E+02 | 3.57E+00 | 5.78E+02 | [M-H]- | C12628 | Vitexin 2''-O-beta-L-rhamnoside | Flavone C-glycosides |
| pmb2976 | 7.55E+02 | 3.11E+02 | 3.64E+00 | 7.56E+02 | [M-H]- | - | Chrysoeriol C-pentosyl-O-hexosyl-O-hexoside | Flavone C-glycosides |
| pmb0565 | 5.09E+02 | 3.48E+02 | 3.36E+00 | 5.08E+02 | [M+H]+ | - | Syringetin 3-O-hexoside | Flavonol |
| pme2898 | 3.21E+02 | 1.53E+02 | 3.53E+00 | 3.20E+02 | [M+H]+ | C02906 | Dihydromyricetin | Flavonol |
| pme1606 | 5.95E+02 | 2.87E+02 | 3.83E+00 | 5.94E+02 | [M+H]+ | - | Kaempferol 3-O-robinobioside (Biorobin) | Flavonol |
| pme0370 | 5.95E+02 | 2.87E+02 | 3.83E+00 | 5.94E+02 | [M+H]+ | - | Kaempferol 3-O-rutinoside (Nicotiflorin) | Flavonol |
| pma0214 | 4.79E+02 | 3.17E+02 | 3.84E+00 | 4.78E+02 | [M+H]+ | - | methylQuercetin O-hexoside | Flavonol |
| pme3130 | 4.65E+02 | 3.03E+02 | 3.85E+00 | 4.64E+02 | [M+H]+ | - | Quercetin 4'-O-glucoside (Spiraeoside) | Flavonol |
| pme3268 | 4.49E+02 | 2.87E+02 | 3.93E+00 | 4.48E+02 | [M+H]+ | C12626 | Kaempferol 3-O-galactoside (Trifolin) | Flavonol |
| pme0361 | 4.35E+02 | 3.03E+02 | 3.96E+00 | 4.34E+02 | [M+H]+ | - | Quercetin 3-alpha-L-arabinofuranoside (Avicularin) | Flavonol |
| pma1116 | 3.01E+02 | 2.59E+02 | 3.98E+00 | 3.00E+02 | [M+H]+ | C10098 | Kaempferide | Flavonol |
| pmb0595 | 4.79E+02 | 3.18E+02 | 4.00E+00 | 4.78E+02 | [M+H]+ | - | Isorhamnetin 5-O-hexoside | Flavonol |
| pme1524 | 3.05E+02 | 1.53E+02 | 4.08E+00 | 3.04E+02 | [M+H]+ | C01617 | Dihydroquercetin (Taxifolin) | Flavonol |
| pma6639 | 4.79E+02 | 3.18E+02 | 4.11E+00 | 4.78E+02 | [M+H]+ | - | Isorhamnetin O-hexoside | Flavonol |
| pme1480 | 3.19E+02 | 3.19E+02 | 4.39E+00 | 3.18E+02 | [M+H]+ | C10107 | Myricetin | Flavonol |
| pme2954 | 3.03E+02 | 3.03E+02 | 5.08E+00 | 3.02E+02 | [M+H]+ | C00389 | Quercetin | Flavonol |
| pme0200 | 2.87E+02 | 2.87E+02 | 5.69E+00 | 2.86E+02 | [M+H]+ | C05903 | Kaempferol | Flavonol |
| pme1502 | 3.15E+02 | 3.00E+02 | 7.17E+00 | 3.14E+02 | [M+H]+ | - | Kumatakenin | Flavonol |
| pme3137 | 2.39E+02 | 1.65E+02 | 7.65E+00 | 2.38E+02 | [M+H]+ | C20871 | 3-Hydroxyflavone | Flavonol |
| pme3484 | 4.79E+02 | 3.17E+02 | 3.43E+00 | 4.80E+02 | [M-H]- | - | Myricetin 3-O-galactoside | Flavonol |
| pme0197 | 6.09E+02 | 6.09E+02 | 3.62E+00 | 6.10E+02 | [M-H]- | C05625 | Quercetin 3-O-rutinoside (Rutin) | Flavonol |
| pme1539 | 6.23E+02 | 3.14E+02 | 3.75E+00 | 6.24E+02 | [M-H]- | - | Isorhamnetin 3-O-neohesperidoside | Flavonol |
| pme3393 | 2.87E+02 | 1.35E+02 | 3.57E+00 | 2.88E+02 | [M-H]- | C01378 | Fustin | Flavonol |
| pme2491 | 5.78E+02 | 4.31E+02 | 3.77E+00 | 5.78E+02 | [M-H]- | C16981 | "Kaempferol 3,7-dirhamnoside (Kaempferitrin)" | Flavonol |
| pme3211 | 4.63E+02 | 3.01E+02 | 3.81E+00 | 4.64E+02 | [M-H]- | C05623 | Quercetin 3-O-glucoside (Isotrifoliin) | Flavonol |
| pme3442 | 4.77E+02 | 3.01E+02 | 3.67E+00 | 4.78E+02 | [M-H]- | - | Quercetin 7-O-β-D-Glucuronide | Flavonol |
| pmb3026 | 5.05E+02 | 3.01E+02 | 3.91E+00 | 5.06E+02 | [M-H]- | - | Quercetin O-acetylhexoside | Flavonol |
| pme1622 | 4.47E+02 | 2.87E+02 | 4.03E+00 | 4.48E+02 | [M-H]- | C12249 | Kaempferol 3-O-glucoside (Astragalin) | Flavonol |
| pme3296 | 4.31E+02 | 2.85E+02 | 4.45E+00 | 4.32E+02 | [M-H]- | C16911 | Kaempferol 3-O-rhamnoside (Kaempferin) | Flavonol |
| pme2963 | 2.87E+02 | 1.25E+02 | 4.58E+00 | 2.88E+02 | [M-H]- | C00974 | Aromadedrin (Dihydrokaempferol) | Flavonol |
| pme0321 | 4.31E+02 | 2.85E+02 | 4.72E+00 | 4.32E+02 | [M-H]- | - | Kaempferol 7-O-rhamnoside | Flavonol |
| pme2973 | 6.75E+02 | 5.13E+02 | 4.83E+00 | 6.76E+02 | [M-H]- | C17555 | "Icariin (kaempferol 3,7-O-diglucoside 8-prenyl derivative)" | Flavonol |
| pme3407 | 3.31E+02 | 3.16E+02 | 5.10E+00 | 3.32E+02 | [M-H]- | C12633 | Laricitrin | Flavonol |
| pme3514 | 3.01E+02 | 1.51E+02 | 5.08E+00 | 3.02E+02 | [M-H]- | C10105 | Morin | Flavonol |
| pme3401 | 3.45E+02 | 3.30E+02 | 5.59E+00 | 3.46E+02 | [M-H]- | C11620 | Syringetin | Flavonol |
| pme1588 | 3.15E+02 | 3.00E+02 | 5.81E+00 | 3.16E+02 | [M-H]- | C10084 | Isorhamnetin | Flavonol |
| pmb3894 | 3.29E+02 | 3.14E+02 | 5.86E+00 | 3.30E+02 | [M-H]- | - | Di-O-methylquercetin | Flavonol |
| pme3369 | 3.15E+02 | 1.65E+02 | 6.19E+00 | 3.16E+02 | [M-H]- | C10176? | Rhamnetin (7-O-methxyl quercetin) | Flavonol |
| pme3288 | 3.29E+02 | 3.14E+02 | 6.32E+00 | 3.30E+02 | [M-H]- | C01265 | "3,7-Di-O-methylquercetin" | Flavonol |
| pmb0723 | 6.89E+02 | 3.30E+02 | 4.92E+00 | 6.88E+02 | [M+H]+ | - | Tricin 4'-O-(β-guaiacylglyceryl) ether O-hexoside | Flavonolignan |
| pmb0743 | 5.27E+02 | 3.32E+02 | 5.38E+00 | 5.26E+02 | [M+H]+ | - | Tricin 7-O-β-guaiacylglycerol | Flavonolignan |
| pmb0746 | 5.27E+02 | 3.32E+02 | 5.79E+00 | 5.26E+02 | [M+H]+ | - | Tricin 4'-O-β-guaiacylglycerol | Flavonolignan |
| pmb1466 | 5.11E+02 | 3.32E+02 | 6.28E+00 | 5.10E+02 | [M+H]+ | - | Tricin 4'-O-syringic acid | Flavonolignan |
| pmb3047 | 6.57E+02 | 4.95E+02 | 4.51E+00 | 6.58E+02 | [M-H]- | - | Tricin 4'-O-(syringyl alcohol) ether 5-O-hexoside | Flavonolignan |
| pmb3049 | 6.57E+02 | 3.29E+02 | 4.65E+00 | 6.58E+02 | [M-H]- | - | Tricin 4'-O-(syringyl alcohol) ether 7-O-hexoside | Flavonolignan |
| pme3261 | 2.71E+02 | 2.15E+02 | 4.32E+00 | 2.70E+02 | [M+H]+ | C14314 | 6-Hydroxydaidzein | Isoflavone |
| pme3266 | 2.71E+02 | 2.01E+02 | 4.37E+00 | 2.70E+02 | [M+H]+ | C02495 | 2'-Hydroxydaidzein | Isoflavone |
| pme3400 | 4.47E+02 | 2.85E+02 | 5.18E+00 | 4.46E+02 | [M+H]+ | C05376 | Sissotrin | Isoflavone |
| pme1498 | 2.69E+02 | 2.69E+02 | 6.27E+00 | 2.68E+02 | [M+H]+ | C00858 | Formononetin (4'-O-methyldaidzein) | Isoflavone |
| pme3208 | 4.45E+02 | 2.82E+02 | 3.51E+00 | 4.46E+02 | [M-H]- | C16195 | Glycitin | Isoflavone |
| pme3209 | 4.31E+02 | 2.69E+02 | 3.93E+00 | 4.32E+02 | [M-H]- | C09126 | Genistein 7-O-Glucoside (Genistin) | Isoflavone |
| pme3502 | 4.29E+02 | 2.67E+02 | 4.54E+00 | 4.30E+02 | [M-H]- | C10509 | Formononetin 7-O-glucoside (Ononin) | Isoflavone |
| pmb0837 | 5.77E+02 | 4.25E+02 | 2.87E+00 | 5.76E+02 | [M+H]+ | - | Procyanidin A3 | Proanthocyanidins |
| pme0436 | 5.77E+02 | 4.07E+02 | 2.87E+00 | 5.78E+02 | [M-H]- | - | Procyanidin B3 | Proanthocyanidins |
| pme0434 | 5.77E+02 | 4.07E+02 | 2.87E+00 | 5.78E+02 | [M-H]- | - | Procyanidin B2 | Proanthocyanidins |
| pme0430 | 5.75E+02 | 2.85E+02 | 3.69E+00 | 5.76E+02 | [M-H]- | - | Procyanidin A1 | Proanthocyanidins |
| pme0432 | 5.75E+02 | 2.85E+02 | 3.64E+00 | 5.76E+02 | [M-H]- | C10237 | Procyanidin A2 | Proanthocyanidins |
